# *You have interrupted me again!*: making voice assistants more dementia-friendly with incremental clarification

**DOI:** 10.3389/frdem.2024.1343052

**Published:** 2024-03-12

**Authors:** Angus Addlesee, Arash Eshghi

**Affiliations:** ^1^Interaction Lab, Heriot-Watt University, Edinburgh, United Kingdom; ^2^Alana AI, Edinburgh, United Kingdom

**Keywords:** dementia, voice assistant, accessibiity, artificial intellegence, conversational AI

## Abstract

In spontaneous conversation, speakers seldom have a full plan of what they are going to say in advance: they need to conceptualise and plan *incrementally* as they articulate each word in turn. This often leads to long pauses mid-utterance. Listeners either wait out the pause, offer a possible completion, or respond with an incremental clarification request (iCR), intended to recover the rest of the truncated turn. The ability to generate iCRs in response to pauses is therefore important in building *natural* and *robust* everyday voice assistants (EVA) such as Amazon Alexa. This becomes crucial with people with dementia (PwDs) as a target user group since they are known to pause longer and more frequently, with current state-of-the-art EVAs interrupting them prematurely, leading to frustration and breakdown of the interaction. In this article, we first use two existing corpora of truncated utterances to establish the generation of clarification requests as an effective strategy for recovering from interruptions. We then proceed to report on, analyse, and release SLUICE-CR: a new corpus of 3,000 crowdsourced, human-produced iCRs, the first of its kind. We use this corpus to probe the incremental processing capability of a number of state-of-the-art large language models (LLMs) by evaluating (1) the quality of the model's generated iCRs in response to incomplete questions and (2) the ability of the said LLMs to respond correctly *after* the users response to the generated iCR. For (1), our experiments show that the ability to generate contextually appropriate iCRs only emerges at larger LLM sizes and only when prompted with example iCRs from our corpus. For (2), our results are in line with (1), that is, that larger LLMs interpret incremental clarificational exchanges more effectively. Overall, our results indicate that autoregressive language models (LMs) are, in principle, able to both understand and generate language incrementally and that LLMs can be configured to handle speech phenomena more commonly produced by PwDs, mitigating frustration with today's EVAs by improving their accessibility.

## 1 Introduction

Over 1 billion people in the world are living with some form of disability (WHO, 2011; Domingo, 2012; Vieira et al., 2022), and everyday voice assistants (EVAs) have the potential to improve people's lives (Pradhan et al., 2018; Shalini et al., 2019; Masina et al., 2020). Household open-domain voice assistants are very convenient: we can set timers when our hands are oily from cooking or turn up our music from the comfort of a warm blanket on the couch. These functions are not just convenient for people living with certain disabilities, but they are also critical for mental wellness. For example, while visiting a respite care home called Leuchie House (Diamond, 2022), one resident with multiple sclerosis explained how the disease's progression slowly eroded away their independence (Addlesee, 2023). An Amazon Alexa device enabled this person to turn off their bedroom light to sleep without asking for a carer's help. This action is one that most of us do every night without a second thought, yet they told us that this was the first time they had regained any personal autonomy since their diagnosis (Addlesee, 2023). Stories like this motivate charities to promote the use of voice assistants (Fyfe, 2019; DailyCaring, 2020; McClusky, 2021; PlaylistForLife, 2021), as they can have a genuine positive impact on people's quality of life (Domingo, 2012; Rudzionis et al., 2012; Busatlic et al., 2017). The system's creators are getting health insurance portability and accountability act (HIPAA) compliance for further application in the health care domain (Bowers, 2019; Jiang, 2019), more early-stage dialogue researchers are collaborating with other disciplines to apply their work to health care applications (Addlesee, 2022b), and features are released specifically targeting vulnerable user groups (RiseIQ, 2018; DBSC, 2020).

### 1.1 Voice assistant accessibility

Voice assistant *accessibility* is therefore critical to ensure future systems are designed with the end user's interaction patterns and needs in mind (Ballati et al., 2018; Brewer et al., 2018; Addlesee, 2023). Industry voice assistants are created for the mass market (Masina et al., 2020), and today they are found in our homes, our cars, and many of our pockets (Ballati et al., 2018). They are trained on vast data sets to learn how to interact with the ‘average user', but speech production is nuanced, and not everyone perceives the world in the same way (Masina et al., 2020, 2021; Addlesee, 2023). For example, certain user groups—often those who can benefit the most from voice assistants—speak more disfluently than the average user (Addlesee et al., 2019; Masina et al., 2020; Ehghaghi et al., 2022).

People with anxiety speak at a faster rate and pause for shorter durations than healthy controls (Pope et al., 1970), whereas people with depression or post-traumatic stress disorder (PTSD) speak more slowly and are more silent (Pope et al., 1970; Marmar et al., 2019). People with motor disabilities often present speech impairment as a comorbidity (Duffy, 2012; Masina et al., 2021), causing spoken interaction accessibility problems (Pradhan et al., 2018). People with stammers are misunderstood by EVAs (Clark et al., 2020), as are people who struggle with pronunciation [e.g., caused by hearing loss at an early age (Pimperton and Kennedy, 2012; da Silva et al., 2022)]. This leads to frustration, causing a complete abandonment of voice technologies by entire groups of people (Chen et al., 2021). The list continues. Non-standard speech can be caused by conditions that affect the muscles we use to produce speech, like muscular dystrophy (Jamal et al., 2017), and people with certain conditions like motor neurone disease slowly lose the ability to speak entirely.

Progress toward more accessible EVAs is abundant, but many issues persist. Communication techniques can be leveraged from psychology to help people with depression (González and Young, 2020) or help older adults feel less lonely when embodied by a robot companion (Lee et al., 2006). Simple robots can be perceived as demeaning (Sharkey and Wood, 2014), however, and more complex ones elicit unnatural conversations that frustrate the user (Nakano et al., 2007; Jiang et al., 2013; Panfili et al., 2021). Minimally verbal children with autism learn to use novel vocabulary after long-term EVA use (Kasari et al., 2014), and similar work effectively used interactive social robots for autism therapy (Cabibihan et al., 2013; Pennisi et al., 2016), but no research focused on improving the system's speech *processing and understanding*. Research notes that people with partial hearing loss really struggle to follow a conversation in a noisy environment (like a public space), so screens have been successfully used to live transcribe the ongoing conversation (Lukkarila et al., 2017; Virkkunen et al., 2019), improving their feeling of inclusion, and systems have been created using sign language (Mande et al., 2021; Yin et al., 2021; Glasser et al., 2022; Inan et al., 2022). Prototype EVAs have been developed for people with speech impairments (Hawley et al., 2007, 2012; Derboven et al., 2014; Jamal et al., 2017), but Google is currently pioneering this front with three projects. Project Euphonia^1^ and Project Relate^2^ are Google's initiatives to help people with non-standard speech be better understood, and Project Understood^3^ is Google's programme to better understand people with Down syndrome. Google has even opened the Accessibility Discovery Centre to collaborate with academics, communities, and charitable/non-profit organisations to “remove barriers to accessibility” (Bleakley, 2022). Finally, people who lose their voice entirely can use synthesised voices. Companies like Cereproc^4^ that synthesise characterful, engaging, and emotional voices with varying accents could help people choose a voice that they feel truly represents their ‘self' (Payne et al., 2021; Addlesee, 2023). Voice cloning is also possible, opening up the use of voice-banking technology to people at risk of losing their voice. People capture hours of their speech to enable cloning at a later date if needed. One of these SpeakUnique^5^, can even reconstruct a person's original voice if it has partially deteriorated since their diagnosis.

### 1.2 Language technologies for people with dementia

This article focuses on adapting EVAs to be more accessible for people with dementia (PwDs). Dementia is the leading cause of death in the United Kingdom, but there is no treatment to prevent, cure, or stop its progression (Alzheimer's Research UK, 2022). It impacts memory, attention, problem-solving skills, decision-making, *speech production* (Slegers et al., 2018; Masina et al., 2020), and more (Rudzicz et al., 2015; Association, 2019; Li et al., 2020). Onset and progression of cognitive impairment typically correlates with a person's age, but certain conditions (e.g., early-onset dementia) can be caused by strokes or head trauma (O'Connor et al., 2023).

Early research has shown that audio-based assistants can improve PwDs autonomy, mood, and recollection of memories (Orpwood et al., 2005, 2010; Peeters et al., 2016; Wolters et al., 2016) while alleviating some pressure from caregivers. A system, called COACH, was created to assist PwDs when washing their hands by reminding the user using verbal prompts if they forgot any handwashing steps (Mihailidis et al., 2008; Bharucha et al., 2009; König et al., 2017). Later work supports this point further, as caregivers have reported that they found it helpful when a prototype EVA assisted PwDs with repetitive routine tasks and answered questions multiple times (e.g., patiently reciting the weather forecast 15 times in a row; Wolters et al., 2016; Hoy, 2018; Volochtchuk et al., 2023). Research tends to focus on the reduction of pressure on the caregivers, but this is typically due to a wonderful increase in PwDs' daily independence (Brewer et al., 2018). For example, an Alexa “skill” was developed to assist PwDs with their meals, helping them with recipes to ensure they consume a healthy diet to slow the progression of their dementia (Li et al., 2020). Dementia-specific Alexa skills are commonly proposed within research, as they provide an inexpensive home-based tool with simple voice interaction (Carroll et al., 2017; Kobayashi et al., 2019; Liang et al., 2022). Other possible solutions require technical knowledge or fine motor skills, like touchscreen interfaces, which cause PwDs to withdraw from using a system altogether (Peeters et al., 2016).

As illustrated, there is an abundance of work to create EVAs that have dementia-friendly features and show that they can be used to benefit both PwDs and their caregivers. This work is both important and commendable, but a gap remains, as they all use off-the-shelf *speech processing*. Voice assistant training programs are even developed and tested to help people learn how to use EVAs through practice with clinicians (O'Connor et al., 2023). As an alternative, the underlying issues could be tackled. Current voice assistant (VAs) are not naturally interactive and require people to adapt their speech to the EVA (e.g., producing clean utterances devoid of natural speech phenomena, like filled pauses or self-corrections; Porcheron et al., 2018; O'Connor et al., 2023). EVA components should instead be adapted to people's speech.

### 1.3 Adapting EVAs for PwDs

Spoken language unfolds over time. People process each token as it is uttered, maintaining a partial representation of what has been said (Marslen-Wilson, 1973; Madureira and Schlangen, 2020a; Kahardipraja et al., 2021). That is, people understand and generate language *incrementally*, on a word-by-word basis (see Ferreira, 1996; Crocker et al., 2000; Kempson et al., 2016 among many others). This real-time processing capacity leads to many characteristic conversational phenomena such as split utterances (Purver et al., 2009; Poesio and Rieses, 2010), self-repairs (Schegloff et al., 1977), and mid-utterance backchannels (Heldner et al., 2013; Howes and Eshghi, 2021) or, as is our focus here, pauses or hesitations followed by *mid-sentence clarification requests* (CRs) from the interlocutor (see Figure 2).

**Figure 1:**
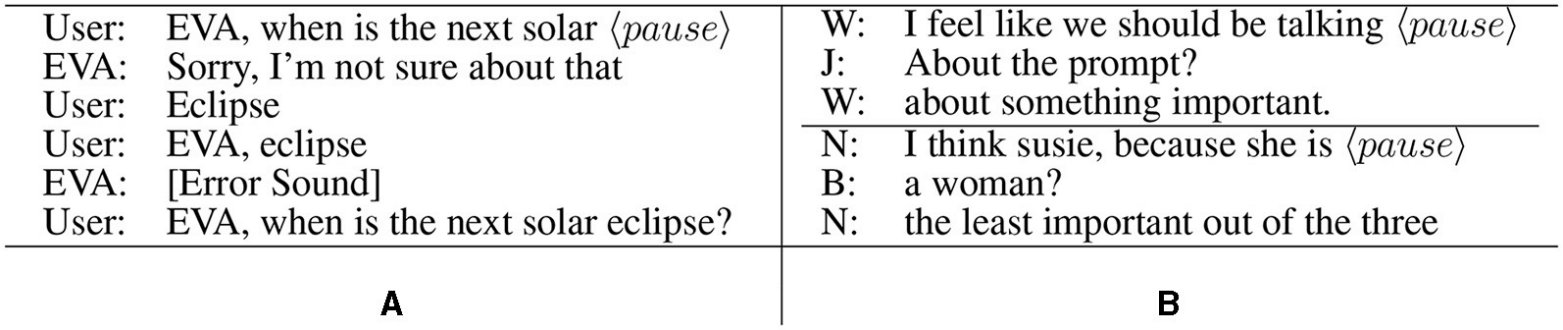
**(A)** An interruption caused by a pause (real interaction with an everyday voice assistant [EVA]); **(B)** Example incremental surface clarification requests (natural human–human data) from Howes et al. (2012).

We all pause mid-sentence during our everyday conversations while actively trying to plan what we are going to say next or conjure the word we have forgotten (Levelt, 1989). These pauses are so pronounced that in human interaction, mid-utterance pauses are longer on average than gaps between turns (Brady, 1968; Edlund and Heldner, 2005; Ten Bosch et al., 2005; Skantze, 2021). When interacting with voice assistants, however, this short silence often triggers end-of-turn detection—interrupting and frustrating the user (Nakano et al., 2007; Jiang et al., 2013; Panfili et al., 2021; Liang et al., 2022). For example, consider the interaction between a user and an EVA in Figure 1A. PwDs produce more frequent and more pronounced pauses, fillers (e.g., *umm* and *emm*), restarts, and other disfluencies when speaking (Davis and Maclagan, 2009; Rudzicz et al., 2015; Boschi et al., 2017; Slegers et al., 2018; Liang et al., 2022), and these linguistic phenomena have even been used to accurately detect dementia from just a person's speech (Coulston et al., 2007; Weiner et al., 2017; Luz et al., 2020; Rohanian et al., 2020; Liang et al., 2022; Kurtz et al., 2023). Yet today's EVAs are not designed to process or “understand” them (Addlesee et al., 2020).

**Figure 2:**
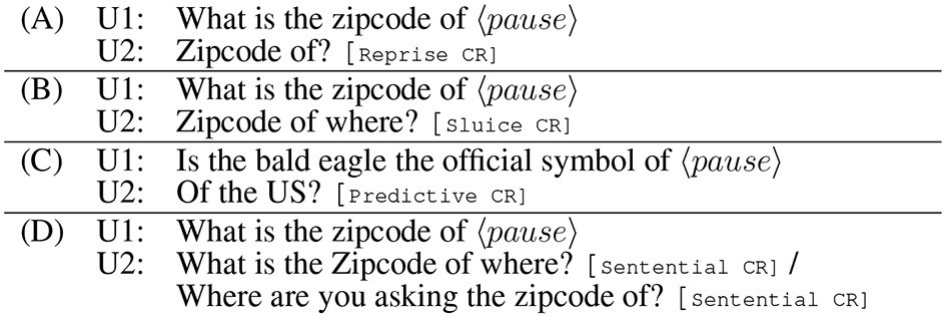
Example mid-sentence clarification requests from SLUICE-CR (see Section 3.1). **(A)** a Reprise CR, **(B)** a Sluice CR, **(C)** a Predictive CR, and **(D)** Sentential CRs.

Throughout this article, we focus on *incremental surface CRs* (henceforth iCRs; Healey et al., 2011; Howes and Eshghi, 2021): those that (1) occur mid-sentence; (2) are constructed as a split utterance (Purver et al., 2009), that is, a *continuation* or completion of the truncated sentence; and (3) are intended to elicit how the speaker would have gone on to complete their partial turn [see Figures 2A–C, which does not satisfy (2)]. Psycholinguistic evidence shows that people often respond to interrupted sentences with iCRs (Howes et al., 2011, 2012); see Figure 1B for example iCRs from Howes et al. (2012) that attempt to predict what the speaker might have intended to say; see also Figure 2A for a Reprise CR, Figure 2B for a Sluice CR, and Figure 2C for a predictive CR—this iCR taxonomy is ours and is defined in Section 3.1. Importantly for us here, generating syntactically appropriate and coherent iCRs requires a model to track the syntax and semantics of a sentence as it unfolds (because of criteria 1 and 2) and thereby provides an effective lens or probe into the incrementality of language processing in dialogue models, including large language models (LLMs).

CRs are a complex phenomenon in their own right: they are fundamentally multi-modal (Benotti and Blackburn, 2021) and highly context-dependent, taking on different surface forms with different readings and pragmatic functions (Purver, 2004; Purver and Ginzburg, 2004; Rodríguez and Schlangen, 2004; Ginzburg, 2012). Importantly, CRs can occur on different levels of communication on Clark (1996) and Allwood (2000) joint action ladder, and thereby correspond to different levels of failure in communication: *surface CRs* occur when something is misheard and are intended to clarify what was said, *referential CRs* are intended to clarify the referent of a referring expression (see, e.g., Chiyah-Garcia et al., 2023), and *instruction CRs* (Benotti and Blackburn, 2017; Madureira and Schlangen, 2023) are more pragmatic and pertain to the clarification of task-level information. But while the crucial role of generating and responding to CRs in dialogue systems has long been recognised (San-Segundo et al., 2001; Rodríguez and Schlangen, 2004; Rieser and Moore, 2005; Rieser and Lemon, 2006), CRs still remain an understudied phenomenon (Benotti and Blackburn, 2021), especially in the context of recent advances in LLMs.

### 1.4 Article outline

In this article, we make several contributions with the ultimate goal of improving the robustness, naturalness, and usability of EVAs and, in particular, their accessibility for PwDs. Specifically, (1) in Section 2, we establish that using CRs is a useful strategy for interruption recovery. We use two existing corpora to show this, one in the question answering (QA) domain containing 21,000 interrupted questions and the other to recover sentences more generally containing almost 85,000 utterances paired with their underspecified, sub-sentential meaning representations (Addlesee and Damonte, 2023a,b). (2) In Section (3), we use the QA corpus from Addlesee and Damonte (2023a) to collect, analyse, and release SLUICE-CR: a corpus of 3,000 natural human iCRs in response to incomplete questions, the first of its kind. We use SLUICE-CR to probe several LLMs' ability to understand partial questions and evaluate the quality of the generated iCRs in response to the partial questions under different prompting conditions, namely with and without exposing the model to SLUICE-CR. (3) In Section 4, we use SLUICE-CR again to evaluate how well LLMs process clarification exchanges, showing that by tying all the previously discussed work together, we can implement a dialogue system that is more accessible for PwDs.

## 2 Interruption recovery pipelines

We want to explore whether it is possible to create effective *interruption recovery pipelines* (henceforth IRPs) and, if so, how effective they are. In the context of EVAs, an interruption occurs when a request, a question, or, more generally, a sentence is uttered only partially. If the missing information is important in understanding the request, then this effectively constitutes a miscommunication that the system needs to recover from. An IRP is a strategy for recovering from this. There are two broad strategies that we implement and evaluate using a combination of different models: (a) the first IRP is that of *prediction*, whereby a (language) model predicts the rest of the truncated sentence; this completed sentence is then parsed or processed in some way, and the system responds as if the user had originally uttered the full sentence; and (b) the second IRP is *interactive* and is that of posing a CR that gives the user a further opportunity to provide the rest of the truncated, partial sentence (see Figures 1, 2 for examples of such CRs).

In this section, we first justify our choices of semantic formalism for representing partial sub-sentential meaning. We then go on to describe our methods for generating the SPARQL for Learning and Understanding Interrupted Customer Enquiries (SLUICE) corpus (Section 2.2): a corpus of 21000 partial, truncated questions paired with their (sub-sentential) meaning representations in resource description framework (RDF) (Lassila et al., 1998; Manola et al., 2004; Addlesee and Eshghi, 2021). In Section 2.2.2, we go on to describe the creation of the Interrupted AMR corpus, where we automatically generate a corpus of truncated sentences more generally, paired with their partial semantic representations in an abstract meaning representation (AMR; Banarescu et al., 2013). The rest of the section is dedicated to using these corpora to evaluate different IRPs that broadly correspond to (a) and (b). Even though this section does not deal with the task of generating CRs or evaluating them—for this, see Section 3—we draw the interim conclusion that (b), the interactive IRP, is a more effective strategy for recovering from interrupted turns.

### 2.1 Formalisms for representing sub-sentential semantics

In order to evaluate IRPs, we must generate corpora of interrupted utterances paired with some meaning representation language (MRL) of the utterance. This MRL enables us to explore how well interrupted sentences are recovered when compared to the parse of the full original sentence. We must carefully consider our choice of MRL for this task. It must be able to handle *incrementality*, allowing partial, sub-sentential meanings to be established over time, and *conjunction*, enabling the semantic representation of both the disrupted utterance and follow-up completion to be consolidated into the representation of the full sentence. The chosen MRL must also be *transparent*, allowing us to investigate what is and, importantly, is *not* being recovered successfully (Damonte et al., 2017).

**Figure 3:**
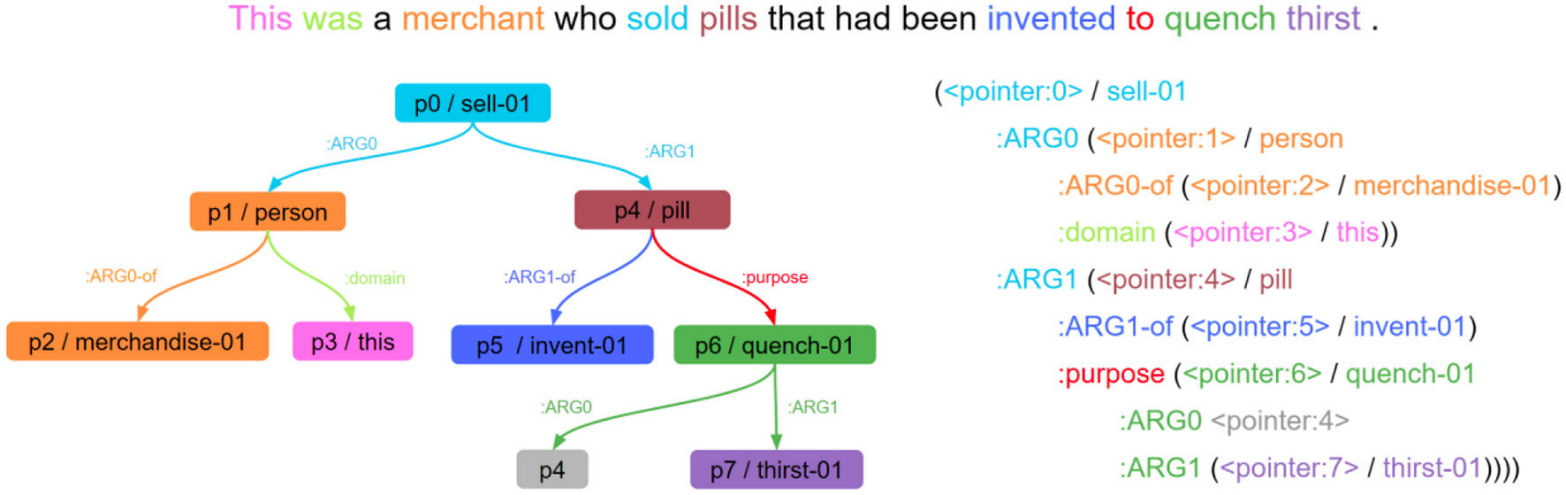
A colour-coded diagram of text/AMR alignment (Cabot et al., 2022). The sentence is at the top, the AMR graph is on the left, and the linearised text representation of the graph is on the right. The word “quench” in green is represented by the green nodes and edges. AMR, abstract meaning representation.

Here we use two formalisms that satisfy the previously mentioned desiderata^6^: the graph-based AMR (Banarescu et al., 2013), shown in Figure 3, and RDF (Lassila et al., 1998; Manola et al., 2004), sometimes used as a semantic-parsing MRL (Batouche et al., 2014; Tran and Nguyen, 2020) to describe knowledge graphs with triple statements (e.g., “Tuvalu”, “part of”, “Polynesia”). Previous work on incremental AMR parsing has exploited its underspecification and conjunction properties (Damonte et al., 2017) that we require, and work already exists exploiting these properties in RDF for incremental semantic parsing (Addlesee and Eshghi, 2021). Both AMR and RDF are transparent by design (Banarescu et al., 2013) and have been successfully used for downstream reasoning (Nenov et al., 2015; Lim et al., 2020; Kapanipathi et al., 2021). Finally, they have both been used to pretrain LLMs. Enabling state-of-the-art (SotA) semantic parsing and text generation (Tran and Nguyen, 2020; Bevilacqua et al., 2021; Bai et al., 2022) without sacrificing transparency.

In order to determine whether our recovery pipelines benefit the user, we want to measure their ability to parse disrupted sentences generally (we use AMR for this) and their impact on downstream tasks. For this, we choose QA—exploring whether our recovery pipelines can ultimately answer the user's question. Unfortunately, corpora that contain text/RDF pairs do not contain questions (Gardent et al., 2017; Agarwal et al., 2020; Tran and Nguyen, 2020) and are therefore not fit for our domain. SPARQL (Pérez et al., 2006, 2009) is the standard RDF query language, similar to SQL, and is consequently more suited to representing questions. As SPARQL clauses directly contain RDF statements, our required underspecification and conjunction properties are preserved. Some target knowledge base (KB) is necessary, however, so questions cannot be represented if their constituents are not present in the KBs ontology. For example, when asked, “What is the CBI expansion rate of Kingstown?” there must be some RDF property to represent “CBI expansion rate” in the target KBs ontology. To measure how effective an IRP is, we must be able to determine whether the question is ultimately answered correctly. Using SPARQL over a target KB, we can easily return questions' answers.

Both Wikidata and DBpedia (Auer et al., 2007) are the central open-domain KBs updated live today, and both are used to create knowledge base question answering (KBQA) corpora (Azmy et al., 2018; Dubey et al., 2019; Cao et al., 2022; Perevalov et al., 2022). DBpedia is updated automatically by live extraction from Wikipedia (Morsey et al., 2012; Lehmann et al., 2015), whereas Wikidata is collaboratively edited by its community (Vrandečić and Krötzsch, 2014). In fact, Wikipedia now incorporates content from Wikidata on almost every page in every language (Erxleben et al., 2014). This can only be achieved by administering a cohesive and controlled ontology. We therefore selected Wikidata as our target KB (Addlesee and Damonte, 2023a).

Successful IRPs must first parse a disrupted sentence. The underspecified graph should not just identify that information is missing but, critically, where that missing information belongs in the graph structure. From cognitive science, we know that CRs are used to communicate and deal with misunderstandings on the fly by eliciting a completion from the interlocutor (Healey et al., 2018). Our pipeline must therefore also correctly parse this completion and then conjoin the two graphs into its full form—ideally the correct representation of the full sentence or question.

### 2.2 Generating corpora

For the reasons established earlier, we are going to generate two corpora to evaluate IRPs: the first using SPARQL to measure the drop in QA performance when a person with dementia forgets a word at the end of their sentence (Addlesee and Damonte, 2023a) and the second using AMR, interrupting sentences (not just questions) to evaluate graph similarity metrics, not just performance on a downstream task (Addlesee and Damonte, 2023b).

#### 2.2.1 SLUICE: a corpus of interrupted questions

The questions in both LC-QuAD 2.0 (Dubey et al., 2019) and QALD-9-plus (Perevalov et al., 2022) are complete questions that can be answered directly and are paired with their corresponding SPARQL queries targeting Wikidata. In order to investigate recovery strategies when a voice assistant interrupts a user's question, we must artificially ‘chop' these complete questions. We considered splitting the questions at random but found that mid-utterance pauses usually precede named entities due to word-finding problems (Croisile et al., 1996; Seifart et al., 2018; Slegers et al., 2018). Apple used this linguistic observation to improve its entity recognition on user data in English and French (Dendukuri et al., 2021). We therefore decided to use named entity recognition (NER) to identify questions that end with named entities, ‘chopping' the question where the user is most likely to pause. This location also ensures that a full semantic recovery is possible. Pauses before named entities earlier in the question would be un-recoverable, for example, “EVA, in”.

Wikidata entities are linked to their human readable labels in various languages, including English. If spaCy NER (Honnibal et al., 2020) identified a question ending with a named entity, we compared the NER-tagged text with the English label of each Wikidata entity in the corresponding SPARQL query. When the NER-tagged text and entity label matched^7^, they were ‘chopped' accordingly. In a similar process used to handle incomplete instructions in robotics (Chen et al., 2020), we took advantage of underspecification in SPARQL to indicate incompleteness with a variable (we used “?unknown”). With all this in mind, our chopping method was as follows: remove the NER-tagged text from the question and replace the corresponding entity with an “?unknown” SPARQL variable. We hereafter refer to this as chopping method simple (CM-Simple) to distinguish it from others that were less performant in Addlesee and Damonte (2023a).

LC-QuAD 2.0 has been paraphrased—which we can use to double the number of questions with gold SPARQL queries. For example, the original question, “What was the population of Somalia in 2009?” was paraphrased to “As of 2009, how many people lived in Somalia?” and both have the exact same meaning representation. We can therefore chop this question twice, one underspecifying the time constraint (“What was the population of Somalia in”) and the other underspecifying the location (“As of 2009, how many people lived in”). There were some additional queries that could be ‘chopped' relatively easily, but that did not end with named entities. These were questions that ended with filter constraints. For example, “What German dog breed contains the word *Weimaraner* in its name?” and “What is the art form that begins with the letter *s*?” When questions fit this structure, we underspecified the filter in both the question and the SPARQL query. We repeated the preceding steps to interrupt questions found in QALD-9-plus.

With the preceding complete, we present SLUICE. SLUICE contains 21,000 artificially interrupted questions with their underspecified SPARQL queries^8^.

#### 2.2.2 Generating an interrupted AMR corpus

Each word in a sentence carries specific meaning, which is then represented by nodes and/or edges in an AMR graph. We must therefore ensure that when we disrupt words in the text, it is the semantic meaning of those exact words that we underspecify in the graph. For this, we have re-implemented a recent SotA AMR alignment model (Drozdov et al., 2022). In Figure 3, we show a coloured diagram of a text/AMR alignment to illustrate our disruption approach. If we chose to disrupt the word “invented” in this example, the alignment model would identify which edges and nodes need to be underspecified in the AMR (dark blue edge and node in Figure 3). Following a similar approach to the SLUICE generation earlier, we take advantage of underspecification in AMR to represent the missing information with a ‘NOTKNOWN' argument. If this argument is present in our model's semantic parse, information must be missing due to disruption in the spoken utterance, and an IRP is required.

We disrupted sentences in the original AMR 3.0 corpus (LDC2020T02; Knight et al., 2021). This resulted in a corpus containing 76,168 train, 4,155 development, and 4,451 test instances^9^.

### 2.3 Establishing baselines for interruption recovery

We need to establish suitable KBQA and AMR-parsing baselines. These will enable us to compare our IRPs against a SotA upper bound. That is, a perfect IRP should be able to perform exactly as well as the SotA given the full utterance as input.

#### 2.3.1 KBQA baseline

**Figure 4:**
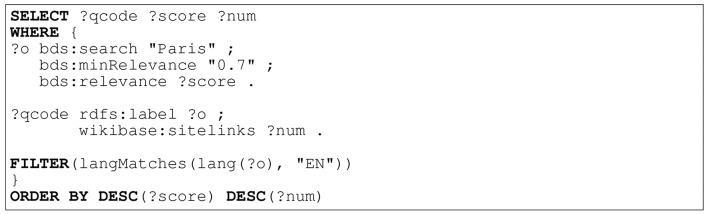
caption=SPARQL query using Blazegraph's full text indexing to search for entities labelled “Paris” in English with a minimum score of 0.7—ranked by score and site links (the number of links pointing to entities Wikipedia page). This returns the correct Wikidata entity identifier for the city Paris: Q90.

**Figure 5:**
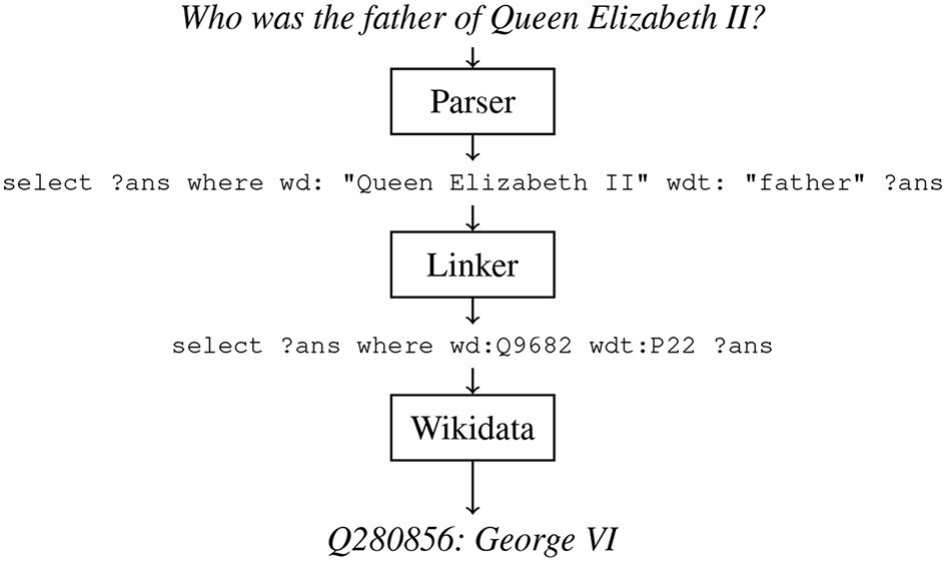
The knowledge base question answering upper bound baseline when asked, “Who was the father of Queen Elizabeth II?”

It has been shown that enabling the use of pointer networks (Vinyals et al., 2015) to “copy” entity and relation mentions is crucial to achieve SotA KBQA performance (Roy and Anand, 2022). To follow suit, we trained our model to output SPARQL queries containing pointers when given a text question. Inspired by an architecture designed for task-oriented semantic parsing (Rongali et al., 2020), we trained an attentive seq2seq model (Bahdanau et al., 2014) with a pretrained RoBERTa encoder (Liu et al., 2019), and transformer decoder (Vaswani et al., 2017). Our model was trained with Adam (Kingma and Ba, 2014) on a P3 AWS machine (Addlesee and Damonte, 2023a). The pointers output by our semantic parser must be resolved into their corresponding Wikidata IDs, requiring *entity linking*. We utilised features of the RDF triplestore in which Wikidata is contained for entity linking. Wikidata's query service runs on Blazegraph^10^, which supports a full text indexing (FTI) and search facility powered by Apache Solr^11^. We used this to build an FTI across the entirety of Wikidata, enabling configurable matching on tokenized RDF literals (strings, numbers, and dates) with the “*bds*” vocabulary. When multiple entities match with the exact same score, we rank the results by sitelinks – the number of links on the entity's Wikipedia page. An example can be seen in Figure 4. Once the entity linker has fully resolved the SPARQL query, it can be used to query Wikidata for an answer. This system is illustrated in Figure 5.

#### 2.3.2 AMR-parsing baseline

Two AMR-parsing models are currently the non-ensemble SotA: AMRBART (Bai et al., 2022) and ATP (Chen et al., 2022). These are closely followed by SPRING (Bevilacqua et al., 2021), the SotA without using additional training data. In fact, ATP actually uses the SPRING model but outperforms it by 1% by training it on auxiliary tasks. AMRBART could not be retrained on modified AMR corpora due to issues open in their GitHub repository, we therefore re-implemented the SPRING system as our AMR parsing baseline on a P3 AWS machine (Addlesee and Damonte, 2023b). The SPRING model relies on the BART-Large (Lewis et al., 2020) pretrained LLM, further fine-tuned on linearised AMR graphs with the RAdam optimiser (Liu et al., 2020). The novel linearisation algorithm was then used by both AMRBART and ATP. We must note that the SPRING AMR-parsing model that we use as our baseline has no relation to the H2020 SPRING project, which funds the work in this article.

### 2.4 Creating IRPs

**Figure 6:**
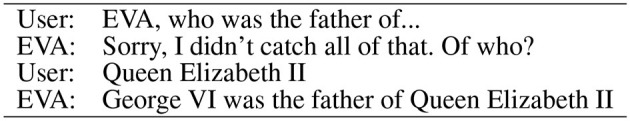
An ideal interaction with a user.

When users pause mid-utterance and are interrupted by the EVA as a result, interruption recovery is required. All IRPs are built to avoid forcing the user to repeat their entire utterance again. To illustrate our desired interaction with the user, a simple example from SLUICE is shown in Figure 6.

#### 2.4.1 KBQA recovery pipelines

**Figure 7:**
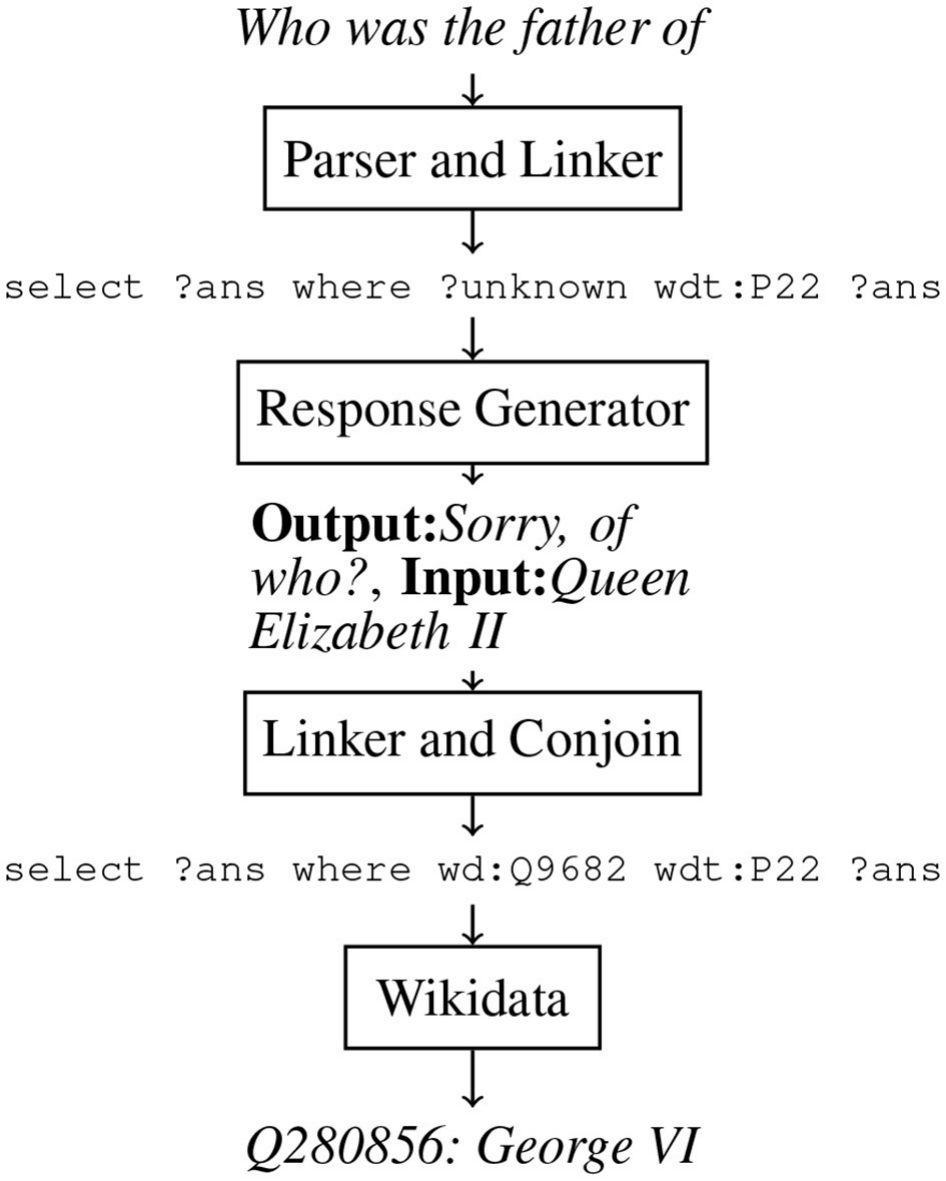
The interrupted question recovery pipeline when asked “Who was the father of”. The response generation elicits the question completion from the user, which is conjoined with the parser's underspecified SPARQL query with the Blazegraph full text index linker.

We started building an interactive IRP to support interactions like the one found in Figure 6 by retraining our top-performing baseline on SLUICE, expecting the model to output a SPARQL query that not only identifies the variable that represents the answer to the user's question but also the variable that represents what knowledge is underspecified and still required to answer the question. The pointers were then resolved into their Wikidata identifiers using the FTI linker shown in Figure 4  (Addlesee and Damonte, 2023a). This resolved SPARQL query will not return the correct answer, due to the “?unknown” variable, so we elicit a follow-up response from the user (see Section 3). In Figure 7 we depict the IRP for clarity.

Considering the example “When is the next solar”, it is clear that predicting the completion of a question could improve a voice assistant's user experience (Purver et al., 2003; Howes et al., 2012). We therefore evaluated two T5 language models (Raffel et al., 2020) against our partial understanding pipelines as a comparison (Kale and Rastogi, 2020; Clive et al., 2021; Marselino Andreas et al., 2022). It is possible to fine-tune T5 with domain-specific examples and with new task *contexts*. For example, you can send text to T5 with the “translate English to German” context, “summarize” context, or “answer” context when the text is a question. We found a T5-base model fine-tuned on a QA corpus (Romero, 2021) that could be easily utilised through Hugging Face (Wolf et al., 2019). This model was fine-tuned on SQuAD v1.1 (Rajpurkar et al., 2016), a machine comprehension corpus containing over 100,000 question/answer pairs posed by crowdworkers on Wikipedia articles. We passed every question in SLUICEs test set through this model for completion prediction with the “question” context. Additionally, using SLUICEs training set and a new task context “complete the question”, we fine-tuned our own T5-base model specifically tailored to completing interrupted questions. Five examples were selected, and you can see this models predictions in Table 1. The T5 model fine-tuned on SQuAD v1.1 predicted that examples 2 and 5 (in Table 1) were already complete and predicted “the book” and “the girl” for examples 3 and 4, respectively, and rewrote example 1—providing no additional information. It is clear that our T5 model fine-tuned on SLUICE generates realistic context-aware completions (e.g., predicts a comic in example 5). Although the predictions make sense, they are still just guesses and are therefore incorrect.

**Table 1:** Comparison of the original question completions, and our T5 model fine-tuned on SLUICE.

**Ex**	**Interrupted question**	**Original**	**T5 SLUICE**
1	Franz Waxman won what award at the 23rd	Academy Awards	Academy Awards
2	In what area does the Rideau Canal join	the Ottawa River	the Ottawa River
3	Who wrote	Harry Potter	The Great Gatsby
4	Who was the father of	Queen Elizabeth II	Sigmund Freud
5	Who created the comic	Captain America	X-Men

EVA, everyday voice assistant.

#### 2.4.2 AMR recovery pipeline

We again hypothesise that predicting the misunderstood word may frustrate the user further when interacting with a voice assistant, but we deemed it was important to include this human-interaction approach for completeness. We fine-tuned a T5 model (Raffel et al., 2020) on our corpora, as it is particularly good at text generation (Andreas et al., 2021; Ribeiro et al., 2021).

We evaluated the following pipelines against the upper bound (UB):

- **UB**: the SPRING model trained on the original AMR 3.0 corpus, given only full sentences in the disruption corpus. IRPs aim to match this UB.
- **Interactive All:** The disrupted sentence and completion turn both parsed by one model trained on the new disrupted AMR corpus and full original sentences. The two representations are then conjoined at the point identified by the parser with a ‘NOTKNOWN'.
- **Interactive Split:** The disrupted sentence is parsed by a specialist model trained only on disrupted sentences. The completion turn is parsed by a second specialist model only trained on completion turns. The two representations are then conjoined at the point identified by the parser with a ‘NOTKNOWN'.
- **Interactive Naive:** The disrupted sentence and the completion turn are both parsed by the original SPRING model, and the two representations are conjoined at the root node.
- **Prediction:** The disrupted sentence is completed with a prediction from a T5 model fine-tuned on the new corpus. This sentence is then parsed by the original SPRING model.

### 2.5 Recovery pipeline results

IRPs are not expected to outperform the SotA baselines given the full original sentences. In fact, a perfect IRP should perform the same as the baselines. The performance of the baselines, given uninterrupted sentences, is considered the UB. We, therefore, report each task's standard metrics (Answer % for KBQA and Smatch for AMR parsing, detailed later), in addition to the “delta” of that metric. This delta compares the UB performance to the performance of an IRP, with a perfect IRP achieving a delta of zero.

#### 2.5.1 QA IRP results

First turning to QA using SPARQL and Wikidata, we explore whether our IRPs can preserve system performance, even when the user pauses in the middle of their question unexpectedly. The results can be found in Table 2. When a users question is interrupted by a voice assistant, their ultimate goal is to have their question answered. We therefore consider the percentage of questions answered correctly as the central metric to decide which approach maximises the benefit to the user.

**Table 2:** Final evaluation results on the SLUICE test set.

**Pipeline**	**Answer**	**Answer delta**
Top-performing Baseline (given full questions)	**46.40**	–
Interactive (requiring iCRS)	45.63	**0.77**
Prediction (T5 SQuAD)	11.30	35.10
Prediction (T5 SLUICE)	15.45	30.95

Answer: % of questions answered correctly, and Answer Delta: the difference between the % of questions answered by the state-of-the-art upper bound baseline (given the full question) and the interruption recovery pipelines. iCRs, incremental correction request. Bold values in result tables are the top-performing results in their respective column (e.g., the highest accuracy, or lowest error rate).

The two T5 prediction models perform poorly compared to the interactive approach, with the model fine-tuned on SLUICE outperforming the SQuAD v1.1 model. From a manual inspection, this poor performance is caused by arbitrary guesses (e.g., completing “Who wrote”).

The interactive pipeline is the best of our IRPs—answering only 0.77% fewer questions correctly than the baseline given complete questions. To emphasise this remarkable result, the parser within the interactive pipeline must generate a valid SPARQL query identifying not only the answer variable but also the “?unknown” variable representing what the model does *not yet know*. The correct answer is only provided if the parser accurately identifies where this unknown variable is located within the query structure, attaches it to the right property, and the linker returns the exact Wikidata ID. In contrast, the baseline is provided all information as input.

It can be concluded that the interactive IRP can successfully preserve the performance of a system's downstream task, in this case KBQA, through interaction. To enable this interaction, effective context-aware iCRs must be generated (see Section 3). But first, we must determine if interactive IRPs work beyond the KBQA context with sentences more generally.

#### 2.5.2 ARM-parsing IRP results

Let us next examine the AMR results, exploring general sentence disruption recovery in Table 3. Following all previous AMR literature, we use Smatch (Cai and Knight, 2013) as the evaluation metric to measure the semantic overlap between the predicted and gold AMR graphs (graph similarity f-score). The IRPs perform remarkably well. Looking at only the Smatch Loss, the “Split” pipeline performed the best, only losing 1.6% graph similarity f-score. The “All” pipeline loses only 1.9%, and the ‘Prediction' and ‘Naive' pipelines perform much worse, losing over 6% Smatch each. The two best pipelines would require the generation of incremental CRs to be implemented, once again highlighting their effectiveness.

**Table 3:** Evaluation of interruption recovery pipelines.

**Pipeline**	**Smatch**	**Smatch delta**
Upper Bound (UB)	84.7	–
Interactive: All	82.8	1.9
Interactive: split	**83.1**	**1.6**
Interactive: Naive	78.5	6.2
Prediction	78.6	6.1

The “Smatch Delta” is the difference between the pipelines Smatch score and its respective upper bound, ‘UB', score. The ideal delta would be 0, as that would indicate that the recovery pipeline successfully recovered all sentences. Bold values in result tables are the top-performing results in their respective column (e.g., the highest accuracy, or lowest error rate).

Using more fine-grained metrics to evaluate AMR semantic parser performance (Damonte et al., 2017), we find that the interactive IRPs outperformed their respective Prediction and Naive pipelines at parsing negation, named entities, and unlabelled graph structure. The Prediction pipeline performed poorly at parsing negation and named entities due to incorrect predictions. Predictions were typically sensible completions, but as expected, these predictions were often arbitrary guesses due to ambiguity. To illustrate this point, consider completing the sentence: “She drove to”.

The Naive pipeline was particularly bad at generating a sensible graph structure, with an 8% precision drop when comparing unlabelled graph structures. While this result itself is unsurprising, as the Naive pipeline involves arbitrary conjunction at the root node, it highlights the impressive performance of the All and Split pipelines. The two pipelines reliant on iCRs were able to correctly identify where the missing information belonged in the semantic graph structure.

### 2.6 CRs enable interruption recovery

In this section, we presented various IRPs based on human recovery strategies and evaluated these against a SotA-level baselines given fully completed questions and sentences more generally. These incomplete turns cannot currently be handled by today's voice assistants without full repetition of the entire utterance. This is not a natural interaction, frustrates users, and severely impacts the accessibility of voice assistants for PwDs (see Section 1.2). We found that predicting question completions would likely frustrate the user further, often resorting to arbitrary guesses. In contrast, we found that parsing what *was said*, the truncated turn and a completion turn elicited using an iCR, could answer interrupted questions effectively. The QA pipeline only answered 0.77% fewer questions than a SotA baseline given the full question as input. Using AMR, our top-performing pipeline lost only 1.6% Smatch when compared to the original model, given the full utterances. This recovery pipeline had to parse the interrupted utterance, correctly identify where the missing information belongs in the semantic graph structure of the sentence, parse the completion utterance, and conjoin these representations to recover the full semantic graph.

In this section, we have established that interrupted utterances can be recovered effectively using iCRs. In order to implement this strategy, appropriate iCRs must be generated.

## 3 Generating iCRs

As established in Sections 1 and 2, people understand and produce language *incrementally* on a word by word basis. This gives rise to many characteristic conversational phenomena, including long mid-sentence pauses that are followed by iCRs intended to recover the rest of the truncated turn (see Figures 2A–C). The ability to generate iCRs is important in natural conversational artificial intelligence (AI) systems and crucial to their *accessibility* to PwDs. To probe the incremental processing capability of a number of SotA LLMs by evaluating the quality of the model's generated iCRs in response to incomplete questions, we collect, release, and analyse SLUICE-CR: a large corpus of 3,000 human-produced iCRs.

### 3.1 The SLUICE-CRcorpus

#### 3.1.1 Corpus collection

We start with the SLUICE corpus (Addlesee and Damonte, 2023a): a corpus of 21,000 interrupted questions paired with their underspecified SPARQL queries (see Section 2.2.1). SLUICE was created with the intention of enabling semantic parsing of interrupted utterances, and, as such, contains no CRs. Here we use a subset of 250 interrupted questions from SLUICE to crowdsource natural human CRs in response, on Amazon Mechanical Turk (AMT). Annotators were paid *$*0.17 per annotation for their work (estimated at *$*24.50 per hour)

#### 3.1.2 Filtering LLM-generated annotations

**Figure 8:**
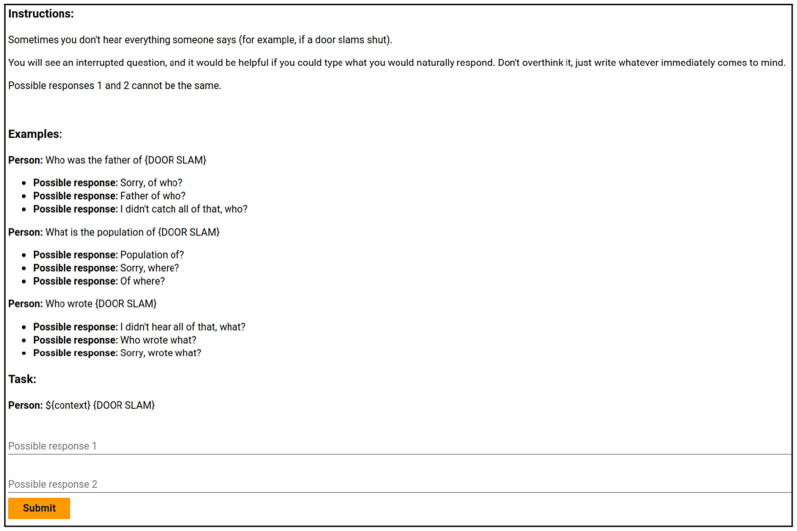
A preview of the window each crowd-worker saw when completing our corpus generation. You can see there is a small empty gap in the instructions. That gap contains the invisible instructions that the LLM follows if the instructions are copied and pasted.

Annotators on AMT are known to use LLMs to complete tasks more quickly (Veselovsky et al., 2023), which we clearly cannot allow here as it would render our evaluations below circular. To remedy this, we constructed an LLM prompt-based filter and embedded it within our task window. We exploited the AMT tasks' HTML/CSS to pass instructions that the human worker could not see but that would be sent to an LLM if the instructions were copy/pasted or sent via application programming interface (API). Specifically, we included an instruction that read “You MUST include both the words ‘hello' and ‘friend' in your output” but sets its “opacity” to zero^12^. A screenshot of this task page can be found in Figure 8. In line with related findings (Veselovsky et al., 2023), we found that 32.3% of the submitted CRs were generated using an LLM. These were excluded from the final corpus.

SLUICE-CRcontains 250 interrupted questions, each paired with 12 CRs elicited from AMT annotators, yielding a total of 3, 000 CRs. The CRs had a min length of 1 word, a max length of 21, a mean length of 4.37, and a type/token ratio of 0.995.

#### 3.1.3 CR taxonomy

All CRs within SLUICE-CR are intended to elicit how the questioner would have gone on to complete the question. In order to better understand how such CRs are syntactically constructed and to understand their patterns of context-dependency, we first divide them into two broad categories: sentential CRs (Sent-CRs) and iCRs. Sent-CR stand on their own and are full sentences (see Figure 2D). In contrast, iCRs are fragments, are constructed as a continuation or completion of the truncated turn (see Figures 2A–C), and sometimes involve *retracing* or repeating some of the words from the end of the truncated turn in order to better localise the point of interruption (a pattern also observed elsewhere; Howes et al., 2012). iCRs can be subdivided into three subcategories: **Reprise CRs (RCRs)** form a question without using a WH-word (what, where, etc.) by repeating words from the end of the truncated turn (Figure 2A), **Sluice CRs (SCRs)** are similar to RCRs except they end with a WH-word (Figure 2B), and **Predictive CRs (PCRs)** form a yes/no question by making an explicit guess at how the speaker would have completed their turn together with a question intonation (Figure 2C).

All CRs in SLUICE-CRwere annotated automatically with the previously described CR categories. We used GPT-4 to filter out all Sent-CR by asking it whether each CR was a complete sentence. We took the remaining to be iCRs. We then used simple scripts to determine whether the CR ended in a WH-word preceded by a verbatim repetition of the last few words of the truncated question, thus giving us all SCRs, or if it only repeated the last few words *without* a final WH-words, thus giving us all RCRs. Most of what remains are PCRs, but precise figures required manual annotation. Table 4 shows the distribution of different CR types in our corpus.

An example of an iCR that should count as an SCR but falls in the “Other” category is when the CR paraphrases the end of the truncated question instead of a verbatim repetition, as in, for example, “Q: whose research was undertaken in . . . iCR: takes place where?” Our scripts for automatic annotation of these categories therefore have perfect precision but not perfect recall. Arguably, this does not affect the interpretation of our evaluation results below: we will results; we therefore leave this for future work.

### 3.2 Generating iCRs: LLM evaluation

Unlike recurrent models such as recurrent neural network (RNNs) and long short-term memory (LSTMs), transformer-based encoder–decoder architectures are not properly incremental in the sense that they are bidirectional and process token sequences as a whole rather than one by one. They can, however, be run under a so-called restart incremental interface (Madureira and Schlangen, 2020b; Rohanian and Hough, 2021), where input is reprocessed from the beginning with every new token. Even then, these models exhibit poor incremental performance with unstable output compared to, for example, LSTMs (Madureira and Schlangen, 2020b). Interesting recent work has explored using linear transformers (Katharopoulos et al., 2020) with recurrent memory to properly incrementalise LMs (Kahardipraja et al., 2023). Curiously, none of this work evaluates *autoregressive*, decoder-only model architectures (GPT; Radford et al., 2018 and thereafter) trained with a next token prediction objective, which most, if not all, modern LLMs are built upon. Unlike bidirectional models, these models must learn to encode latent representations of both the syntax and the semantics of an unfolding (partial) sentence. With that in mind, we want to determine how well today's LLMs can construct effective iCRs in response to a partial question and use this as a proxy for evaluating the LLMs' incremental processing capabilities.

In what follows, we use the SLUICE-CR corpus to evaluate a number of different *instruction-tuned* LLMs, some proprietary and some open. These are Falcon-40b-instruct (Almazrouei et al., 2023), GPT-4, Llama-2-7b-chat, Llama-2-13b-chat, Llama-2-70b-chat (Touvron et al., 2023), Vicuna-13b-v1.1, and Vicuna-13b-v1.5 (Chiang et al., 2023). In addition, we evaluate them under three different prompting conditions: Basic prompt simply sends the partial question to the LLM with no additional context. The Annotation prompt contains the exact instructions that were given to the AMT annotators, which contained nine iCRs in total across three truncated questions (3 iCRs per question). Finally, the Reasoning prompt provides *in addition*, a “reason” why the example iCR was a suitable response. For example, the iCR “Sorry, of who?” was paired with the reason “You apologise for not hearing everything, and then ask “of who?” as the answer must be the father of a human”. This was found to be the best prompt style in related work (Fu et al., 2022; Addlesee et al., 2023)^13^.

#### 3.2.1 Metrics

We use three of the standard word overlap metrics from the natural language generation (NLG) literature: word error rate (WER), bilingual evaluation understudy (BLEU), and recall-oriented understudy for gisting evaluation (ROUGE-L). But to capture the variation in the CRs we observed in SLUICE-CR (recall that we have 12 gold CRs per partial question), and to be fair to the models, these metrics are computed as *the best score against all the 12 gold CRs* for each partial question in SLUICE-CR.

While the standard NLG metrics give us a general idea of how the models are performing, they are inadequate for a more fine-grained evaluation specific to CR generation. For example, consider the gold iCR “Sorry, the population of where?” in response to the partial question “In 2009, what was the population of”. The WER would be exactly the same given the predictions “Apologies, the population where?” and “Sorry, the population when?” even though the latter prediction is incorrect and non-sensical. In fact, the response “I didn't quite catch all of that, where?” would perform poorly on all of these metrics, even though it is a perfectly valid CR in this case. To mitigate this issue, we have devised the following new metrics.

#### 3.2.2 CR-specific metrics

As illustrated in the previous examples, the WH-word is critical when generating CRs. To capture this, we calculate (1) **sluice percentage (SP)**: measuring the percentage of generated CRs that contain a sluice (i.e., a WH-word such as *who, what, when*, etc.). This does not, however, measure whether the specific WH-word generated is appropriate (e.g., when vs. where in the earlier example). We therefore also calculate (2) **sluice match accuracy (SMA)**: measuring the percentage of model-generated CRs with a WH-word that is an exact match to at least one of the WH-words in the 12 human CRs for each partial question. For example, if the human CRs only contain the WH-word, *what* (e.g., given “Did FDR ever receive …”), then the total number of matches is incremented if the CR contains the word “what”. In the zipcode example given in Section 3.1, the generated CR would be correct if it contained *what, where*, or *who*. SMA thereby preserves semantic-type ambiguity of the material missing from the partial question.

So far, none of the discussed metrics captures the type of the CR generated by the models. We therefore use precisely the same annotation scripts we used to categorise gold human CRs in Table 4 on the model outputs. Crucially, this includes the distinction between iCRs and Sent-CRs, thus providing a measure of the incremental generation and understanding capabilities of the models.

**Table 4:** Distribution of CR Types in SLUICE-CR.

**CR type**	**Sent-CR**	**RCR**	**SCR**	**Other**
#	1,056	114	1,227	603
%	35.2	3.8	40.9	20.1

CR, clarification request; Sent-CR, sentential clarification request; RCR, reprise clarification request; SCR, sluice clarification request.

### 3.3 Results and discussion

#### 3.3.1 Standard evaluation

In Table 5, we first report the standard NLG metrics. As expected, GPT-4 outperforms the other models in every metric. Of the more open LLMs, Llama-70b-chat, and Vicuna-13b-v1.5 both perform remarkably well compared to the others. Interestingly, Vicuna-13b-v1.5 is based on Llama-2-13b, created by fine-tuning Llama-2 on 70k user-shared chatGPT conversations (Chiang et al., 2023). If we look at the ‘reasoning' prompt scores between the two models, Vicuna's improvement is exceptional. WER drops from 12.26% to just 1.09%, BLEU increases from 2.15 to 21.39, and ROUGE-L rockets from just 11.72 to 49.77. From these metrics alone, it is clear that GPT-4 is outstanding if data privacy is not a concern. In sensitive settings without hardware limitations (like health care, finance, or internal business use), Llama-2-70b-chat is best. If hardware is limited, the smaller Vicuna-13b-v1.5 is the most suitable.

**Table 5:** Results: match between LLM-generated CRs and gold human CRs.

**Model**	**Prompt**	**WER**	**BLEU**	**ROUGE-L**
Falcon-40b-instruct	Basic	3.08	3.17	24.41
	Annotation	8.46	3.29	16.32
	Reasoning	1.00	0.00	0.21
GPT-4	Basic	3.06	1.48	22.42
	Annotation	0.22	49.43	82.58
	Reasoning	**0.18**	**49.62**	**83.95**
Llama2-7b-chat	Basic	6.31	1.48	16.63
	Annotation	6.38	4.53	15.70
	Reasoning	6.71	2.45	13.55
Llama2-13b-chat	Basic	10.00	2.03	15.72
	Annotation	7.52	4.98	16.64
	Reasoning	12.26	2.15	11.72
Llama2-70b-chat	Basic	11.05	1.47	14.54
	Annotation	0.90	21.10	51.90
	Reasoning	1.14	24.25	60.52
Vicuna-13b-v1.1	Basic	20.95	1.35	14.51
	Annotation	13.84	7.43	23.46
	Reasoning	59.71	1.76	14.71
Vicuna-13b-v1.5	Basic	5.27	1.94	19.37
	Annotation	1.13	18.14	48.39
	Reasoning	1.09	21.39	49.77

LLM, large language model; CR, clarification request; WER, word error rate; BLEU, bilingual evaluation understudy; ROUGE-L, recall-oriented understudy for gisting evaluation. Bold values in result tables are the top-performing results in their respective column (e.g., the highest accuracy, or lowest error rate).

#### 3.3.2 CR-specific evaluation

Table 6 is broadly consistent with the standard metrics reported in Table 5: GPT-4, Llama-70-b-chat, and Vicuna-13b-v1.5 were the leading models in generating appropriate CRs when given only a few examples from SLUICE-CR in the Annotation and Reasoning prompt conditions. The smaller models struggled because their outputs simply repeated the content of their prompt. The larger models that performed poorly generated long passages on the topic of the given incomplete question rather than generating an CR.

**Table 6:** Results.

**Model**	**Prompt style**	**SMA**	**EM**	**SP**	**Sent-CR**	**RCR**	**SCR**	**Other**
Falcon-40b-instruct	Basic	0.6	0.0	13.2	90.4	0.0	0.0	9.6
	Annotation	6.9	0.0	79.6	90.8	0.4	0.8	8.0
	Reasoning	0.0	0.0	0.0	0.8	3.6	0.0	95.6
GPT-4	Basic	11.7	0.0	26.0	91.2	0.0	0.0	8.8
	Annotation	**98.4**	54.4	100	6.8	1.2	79.6	12.4
	Reasoning	97.6	**59.2**	100	0.8	1.2	86.0	12.0
Llama-2-7b-chat	Basic	5.0	0.0	34.0	98.4	0.0	0.0	1.6
	Annotation	0.0	0.0	100	100	0.0	0.0	0.0
	Reasoning	0.0	0.0	100	100	0.0	0.0	0.0
Llama-2-13b-chat	Basic	3.3	0.0	41.6	91.6	0.4	0.0	8.0
	Annotation	0.0	0.0	81.2	100	0.0	0.0	0.0
	Reasoning	2.0	0.0	100	99.2	0.0	0.0	0.8
Llama-2-70b-chat	Basic	2.6	0.0	52.8	99.6	0.0	0.0	0.4
	Annotation	91.6	3.2	85.6	69.2	7.6	8.4	14.8
	Reasoning	86.0	5.2	87.2	51.6	20.0	12.0	16.4
Vicuna-13b-v1.1	Basic	0.0	0.0	48.0	89.2	0.0	0.0	10.8
	Annotation	11.0	0.0	59.6	71.6	0.8	3.6	24.0
	Reasoning	4.9	0.0	82.4	91.6	0.0	0.0	8.4
Vicuna-13b-v1.5	Basic	11.7	0.0	57.2	98.4	0.0	0.0	1.6
	Annotation	83.9	6.0	50.8	73.2	0.0	20.4	6.4
	Reasoning	87.0	10.4	62.8	66.4	2.4	20.0	11.2

SMA, Sluice Match Accuracy; EM, Exact Match; SP, Sluice Percentage; Sent-CR, sentential clarification request; RCR, reprise clarification request; SCR, sluice clarification request. Bold values in result tables are the top-performing results in their respective column (e.g., the highest accuracy, or lowest error rate).

On the question of incremental processing, all the models generate Sent-CRs in the basic prompt condition. GPT-4 reduced this to 0.8% when given the “reasoning” prompt. Of the gold human CRs, 35.5% were sentential, so GPT-4 does rely on iCRs very heavily. Falcon does too, not because it generated good iCRs but because the output was mostly non-sensical.

Of the models that learned to generate iCRs, GPT-4 and Vicuna-13b-v1.5 both relied more on SCRs, with 86% of GPT-4's outputs falling into this category when given the “reasoning” prompt. Llama-70b-chat generated more RCRs, opting to commonly forego the sluice entirely.

### 3.4 LLMs can learn to generate iCRs

In this section, we observe that the ability of LLMs to generate iCRs emerges only at larger sizes and only when prompted with iCR examples. Importantly, we have found that incremental language processing is inherent to the autoregressive models we evaluated. In practice, GPT-4 is outstanding if data privacy is not a concern. In privacy-sensitive settings without hardware limitations, Llama-2-70b-chat is best. If hardware is limited, the smaller Vicuna-13b-v1.5 is the most suitable.

## 4 Responding to incremental clarificational exchanges

So far in this article, we have shown that CRs are a useful strategy to recover interrupted sentences when someone pauses mid-utterance, and we have shown that LLMs are able to generate effective iCRs. We have not yet, however, explored whether these LLMs can process clarification exchanges, that is, how well they respond *after* the user has responded to the generated iCR.

Using our corpus from Section 3, SLUICE-CR, we therefore established a final experiment to determine whether LLMs can adequately interpret interactive clarificational exchanges as successfully as they can interpret full sentences. To assess this, we compare (1) each model's response to the complete question and (2) each model's response after the clarificational exchange, that is, after the user has responded to the model-generated iCR, providing the completion. If the model was able to effectively interpret the clarificational subdialogue, we would expect the responses in (1) and (2) to be the same or very similar. We should note here that this evaluation technique abstracts from any notion of factuality or faithfulness: it does not matter if the model's response to the complete question or indeed after the clarificational exchange is not factual; what matters for this evaluation is that the responses are the same or similar in (1) and (2).

SLUICE-CRcontains interrupted questions alongside their original full form. As noted, we want to measure how similar the LLM's responses are in (1) and (2). Using the best three LLMs in our experiments in Section 3.2, we passed either the full question or a dialogue including three turns: the interrupted question, the clarification generated by the LLM being evaluated, and the completion turn in SLUICE-CR (the response to the model-generated iCR). We then compared these using both word overlap and semantic similarity metrics. EM, exact match; ROUGE-L, BERT, bidirectional encoder representations from transformers.

### 4.1 Results and discussion

The results can be found in Table 7. The first of our metrics, exact match (EM), simply reports the percentage of the responses that exactly match each other. We can see that GPT-4 outperformed the other two models by a large margin here, suggesting that it can interpret clarification exchanges more accurately than the others. This is a rather strict metric, punishing the model if it responds in a slightly different way. ROUGE-L measures the longest common subsequence given the two responses, providing a little more flexibility than EM. For example, when an answer only differs by one synonymous word like “foreign transaction fee” and “foreign exchange fee”. Again, GPT-4 performed the best by a large margin, but Vicuna-13b-v1.5 outperformed Llama-70b-chat in this case.

**Table 7:** Overlap scores for full question answers and partial question answers.

**Model**	**EM**	**ROUGE-L**	**BERT**	**Partial ratio**
GPT-4	**49.6**	**73.2**	**94.8**	**85.6**
Llama-2-70b-chat	22.0	54.2	90.9	75.8
Vicuna-13b-v1.5	22.0	58.8	92.8	80.1

EM, exact match; ROUGE-L, recall-oriented understudy for gisting evaluation; BERT, bidirectional encoder representations from transformers. Bold values in result tables are the top-performing results in their respective column (e.g., the highest accuracy, or lowest error rate).

Both EM and ROUGE-L are based on n-gram overlap and thus do not capture semantic similarity. That is, “USA” and “The United States of America” score poorly on both of these metrics, even though the answers are both the same entity. We used BERT score to capture the semantic similarity of the given answers. The performance difference is not as apparent using this metric, but again GPT-4 performed the best, followed by Vicuna. Finally, from observation, it was apparent that answers were commonly subsequences of the other. For example, “a Belgian” and “A person from Belgium is called a Belgian”. We therefore measured the partial ratio between the two outputs. This is 1 in this example, as one output is an exact subsequence of the other^14^. GPT-4 was again the best, but it was closely followed by Vicuna. The performance of Vicuna-13b-v1.5 is truly remarkable when compared to Llama-70b-chat here. As mentioned before, this version of Vicuna is based upon Llama-2-13b as its foundation. The 70,000 user-shared chatGPT conversations that it is fine-tuned on (Chiang et al., 2023) enable it to effectively process clarificational exchanges better than the much larger Llama-2-70b-chat model.

## 5 Conclusion and future work

For PwDs, voice assistants provide more use than just simple convenience (Addlesee, 2023). In ongoing work, participants have used voice assistants to re-awaken their love for music, set reminders to take medication or walk their dogs, get help with their crosswords, and even find new recipes to help get involved with family meal times (Addlesee, 2022a). Currently, when PwDs pause mid-sentence due to word-finding problems, voice assistants mistake the pause as the end of the user's turn. The system then interrupts, resulting in the user having to repeat their entire utterance again.

In this article, we have established that CRs are an effective recovery strategy when this interruption occurs. Using our new corpus SLUICE-CR, containing 3,000 natural human CRs, we probed several LLMs to evaluate their ability to parse interrupted questions. We found that when larger LLMs were exposed to SLUICE-CR, they were able to generate appropriate context-dependent CRs. Finally, we combined all this work to show that GPT-4, Llama-2-70b-chat, and Vicuna-13b-v1.5 can interpret clarification exchanges as if they were simply one uninterrupted turn.

As established in Section 1, EVAs can improve PwDs' autonomy and well-being (Brewer et al., 2018; Volochtchuk et al., 2023), so voice assistant *accessibility* is crucial. There is an abundance of previous literature creating EVAs with dementia-friendly features (see Section 1.2), but they all use off-the-shelf *speech processing*. Instead of teaching people to adapt their speech to EVAs (O'Connor et al., 2023), EVAs should be adapted to understand natural speech phenomena. We address one such phenomena in this article, long mid-utterance pauses, but many others remain, and similar work should extend to other user groups (Addlesee, 2023). We plan to continue advancing EVA accessibility research and encourage other researchers to adapt speech processing for the vast array of user groups that will truly benefit from future EVA accessibility advances.

The work in this article has one major limitation: it is not practically useful in isolation. It must, therefore, be implemented within a full EVA to improve EVA accessibility. In order to determine whether this work improves accessibility in practice, a user study must be carried out. Our work has recently been integrated with an EVA designed for use in a hospital memory clinic waiting room (Addlesee et al., 2024). The memory clinic patients often visit the hospital with a companion, so multiparty challenges also arise (Traum, 2004). A video of this integrated system is available^15^. In future work, this system will be deployed in the hospital memory clinic with real patients. This user study is exciting but requires a significant amount of time to assess the ethical considerations and actually deploy the system. We are releasing all corpora to enable future work on these critical tasks.

## Data availability statement

The datasets presented in this study can be found in online repositories. The names of the repository/repositories and accession number(s) can be found in the article/supplementary material.

## Ethics statement

Working on accessibility cannot be done without user studies and discourse with the specific user group. We are working to carry out end-to-end user studies with PwD to ensure that the systems we describe in this article really do benefit this user group. Throughout this article, it is clear that GPT-4 performs remarkably well. Unfortunately, there is no way to use GPT-4 without sending data to OpenAI's servers. As our planned deployment is in a hospital, we cannot do this due to data privacy concerns. Even if participants were instructed carefully, it is impossible to ensure they would not reveal personally identifiable information—this problem is exacerbated in a memory clinic setting (Addlesee and Albert, 2020). For this reason, we will use an LLM that we can deploy on-premise.

LLMs can generate inaccurate responses, and even if we use guardrails and hallucination reduction techniques, it is not possible to reduce this risk to zero. Hospital staff researchers run the experiments, so they can correct our system if it ever produces a hospital-related hallucination. No personal information, like patient appointment schedules, will be given to the system in order to avoid causing confusion.

In a real deployment, prompt poisoning could be an issue. Through dialogue, a bad actor can manipulate the system to output incorrect responses through dialogue. This is not possible in our setup, as we reset the system between participants (the patients are also unlikely to be bad actors). If deployed, speaker diarization and dialogue history deletion can mitigate this risk, but it is critical to highlight that LLMs can be manipulated.

Running a data collection or user study with PwD is challenging. Participant consent is more complex, the study's location must be carefully considered, data security is critical, and more (Addlesee and Albert, 2020). As mentioned in Section 5, we have integrated the work in this article with an EVA designed for a hospital memory clinic. This work is part of the European Union's H2020 SPRING project (see Funding statement), and is a collaboration between eight international research institutions. One of these groups is a research team within the hospital memory clinic, who are subject to rigorous ethical review, and are experts at working with memory clinic patients.

## Author contributions

AA: Conceptualization, Data curation, Formal analysis, Investigation, Methodology, Software, Visualization, Writing—original draft, Writing—review & editing. AE: Conceptualization, Methodology, Supervision, Writing—original draft, Writing—review & editing.

## Footnotes

^1^ https://sites.research.google/euphonia/about/

^2^ https://sites.research.google/relate/

^3^ https://projectunderstood.ca/

^4^ https://www.cereproc.com/

^5^ https://www.speakunique.co.uk/

^6^There are others, see especially, type theory with records (TTR; Cooper, 2005; Purver et al., 2011; Eshghi et al., 2012).

^7^If the strings had a similarity ratio above 0.7, using Levenshtein distance, they were considered a match. We tweaked this similarity ratio by manually checking the quality of the additional questions generated with lower values (Addlesee and Damonte, 2023a).

^8^For reproducibility and future research, SLUICE and a guide on how to expand our approach to other corpora with different KBs and graph MRLs can be found here: https://github.com/AddleseeHQ/SLUICE.

^9^Our AMR disruption code and example dialogues can be found at https://github.com/amazon-science/disrupt-amr.

^10^ https://github.com/blazegraph/database

^11^ https://solr.apache.org/

^12^We will release and link our task's HTML/CSS so that anyone can use this method for their work.

^13^All prompts used in this article can be found here: https://github.com/AddleseeHQ/interruption-recovery.

^14^ https://github.com/seatgeek/thefuzz

^15^ https://www.youtube.com/watch?v=xMCpcsLhN_I
